# Impact of occupational heat exposure on selected haematological parameters of bakery workers: A comprehensive study in Ilorin, Kwara State

**DOI:** 10.1097/MD.0000000000036914

**Published:** 2024-01-12

**Authors:** Arinze Favour Anyiam, Onyinye Cecilia Arinze-Anyiam, Ajiboye Mariam Oyindamola, Emmanuel Ifeanyi Obeagu

**Affiliations:** a Department of Medical Laboratory Science, Haematology Unit, Faculty of Basic Medical and Health Sciences, Thomas Adewumi University, Oko, Nigeria; b Department of Medical Laboratory Science, Kampala International University, Ishaka, Uganda.

**Keywords:** bakery workers, erythrocyte sedimentation rate, haematological parameters, heat stress

## Abstract

The Occupational Health and Safety Administration has identified indoor industries at risk of experiencing heat-related illnesses due to the presence of heat-generating appliances; these include bakeries, kitchens, laundries, and furnaces. This study aimed to assess the effects of heat stress on haematological parameters and erythrocyte sedimentation rates (ESR) of bakery workers in Ilorin, Kwara State. It was a cross-sectional study, involving laboratory analysis, which lasted for 3 months. A total of 60 Male and female bakery workers aged between 18 and 65 years with 1 or more years of working experience were recruited for this study. Full blood count estimation was carried out using haematological analyzer (Sysmex-2000) and the ESR was carried out using the Westergren method in the Haematology unit, Kwara State University, Malete. Results were analyzed using SPSS version 20. This study found the bakery workers to have low ESR (2.13 ± 1.28) compared to (10.05 ± 4.95) of the control, the RBC was high (6.708 ± 2.08) compared to (5.46 ± 1.12) of the control group. TWBC was also found to be high (7.425 ± 1.74) compared to (6.95 ± 2.49) of the control population. Findings from this study concluded that working under high temperatures negatively affected the health of bakery workers with reports of heat stress-related symptoms and also affected ESR and haematological parameters. A comprehensive and effective national occupational health and safety program that includes relevant policies, decrees, and proper enforcement is needed to ensure the worker’s safety and health both in the formal and the fast-growing informal sectors.

## 1. Introduction

The issue of occupational heat exposure is becoming increasingly pressing on a global scale due to the effects of climate change.^[1]^ Working in extreme heat (i.e., temperatures above 35°C), whether indoors or outdoors, puts workers’ health at risk and lowers labor productivity.^[2]^ Overexposure to heat is a highly dangerous occupational hazard, especially in indoor and outdoor environments without adequate temperature control.^[3]^ According to estimates, heat exposure costs South East Asian workers 20% of their working hours.^[4]^ In both indoor and outdoor workplaces, projections for 2030 indicate that heat exposure will cost labor productivity by roughly 70 million work-life years and cause occupational heat stroke death to cost 880,000 work-life years.^[5]^

When the body temperature rises above 38°C, heat stress in hot work conditions occurs. This can cause symptoms like chills, dry skin, elevated body temperature, confusion, dizziness, fainting, exhaustion, weakness, nausea, and cramping in the muscles.^[6,7]^ Heat exhaustion, heat syncope, heat cramps, and heat stroke are further heat stress-related medical problems.^[6,8]^ Furthermore, exposure to intense heat has been linked to negative impacts on mental health.^[9]^ According to a 2018 comprehensive review, there is a link between heat exposure and occupational injuries that are primarily brought on by exhaustion, decreased psychomotor function, loss of focus, and decreased alertness.^[10,11]^

Haematological markers are useful for determining a person’s physiological state.^[12]^ Haematology is the study of blood components, including red blood cells, white blood cells, and platelets, and how these characteristics are measured and used to diagnose and track diseases.^[13]^ Haematological investigations help determine the level of blood damage as well as in diagnosing a variety of disorders.^[14]^

The rate at which red blood cells sediment is known as the erythrocyte sedimentation rate (ESR), and it is frequently employed as a generic metric to track disease activity and aid in the diagnosis of numerous inflammatory illnesses.^[15]^ Three phases have been identified for the ESR, which is a poorly understood phenomenon: Red Blood Cell (RBC) aggregation, precipitation, and packing. Sedimentation depends on RBC aggregation, which is made possible by the presence of specific plasma proteins known as agglomerins, such as fibrinogen, IgM, and α2-macroglobulin. The amount and form of red blood cells as well as plasma viscosity are two variables that can alter these three phases, and they can also alter the sedimentation rate. The ESR varies between age groups and sexes and is measured in millimeters per hour.^[15]^

The Westergren method, so named since it was originally described by Westergren and Fahraeus, is a test used to measure the ESR. The approach that was first presented has not undergone significant modifications, and all methods that have been introduced since then have been assessed using the Westergren method as the benchmark. The preferred approach was suggested by the International Council for Standardisation in Haematology to be the Westergren method. Errors in the dilution of blood caused by the Westergren method’s use of citrate, a liquid-based anticoagulant, have a substantial impact on the ESR.^[15]^ Widely used variations of the reference technique, also known as the modified Westergren method, substitute EDTA for citrate as the anticoagulant. Dilution mistakes are decreased by EDTA, a solid-based anticoagulant that dilutes significantly <1%.^[16]^ Recently, several changes have been made to the current process, such as the introduction of automated devices, to reduce human contact with blood products and speed up turnaround times.^[16]^

There has been little research on the effects of heat on indoor workers. To calculate the risk of heat exposure and heat-related illnesses among indoor workers in other professions – bakery workers included – more research is required. Further assessments are required in addition to indoor temperature monitoring. These assessments should include evaluations of personal variables and occupational duties that may influence the dangers associated with indoor heat exposure. This gap made the project work necessary to determine how bakery heat stress affects certain haematological parameters and how heat affects the pace at which red blood cells sediment among bakers in Ilorin, Kwara State when they stand vertically for an hour.

The majority of research on bakery workers has been done on industrialized bakeries, examining the effects of flour dust exposure on the respiratory system as well as skin irritation and allergies. Additional studies and evaluations of ergonomic risks, primarily related to musculoskeletal problems, as well as the mental and self-rated health of bakery employees, have been published. Studies have also evaluated chronic venous illness, oxidative stress, burn injuries, and cataracts brought on by prolonged exposure to kerosene and biomass burners. Nonetheless, the ESR and haematological parameters of bakery employees have received little attention. Therefore, this study will look at how heat affects certain haematological parameters and how heat stress affects how quickly red blood cells sediment after standing upright for one hour.

## 2. Materials and methodology

### 2.1. Study area

This study was carried out in Ilorin, the capital of Kwara State, North Central Nigeria, is a town located between the Northern and Southern parts of Nigeria at a distance of about 302 km north of Lagos and 475 km south of Abuja, the Federal Capital Territory.

### 2.2. Duration of study

This study lasted for 3 months from sample collection to the analysis of the samples.

#### 2.2.1. Study design.

It was a cross-sectional study, involving laboratory analysis.

### 2.3. Ethical consideration

Approval for this study was obtained from the Kwara State Ministry of Health. The sample collection was explained to the patient using the information sheet. Each patient was given a written informed consent before being engaged in this study. Each patient was given informed consent before being recruited to the study.

### 2.4. Sample size determination

Using a convenient sampling technique, Ilorin was divided into geographical sectors East north, south and West. A systematic random sample technique was then used to select 5 bakeries from each geographical region (20 Bakeries). Three bakery workers were selected randomly from the 20 bakeries selected from the 4 geographical regions.

The minimum sample size was 60.

For this study, a total of 60 bakery workers were recruited.

For this study 20 control samples from non-bakery workers were recruited.

### 2.5. Data collection

A semi-structured questionnaire was administered by the researchers to collect necessary data. The questionnaire assessed personal information (age, gender, marital status, educational level, residence), working hours and duration of work (in years), and health-related symptoms. The respondents were also asked about ailments they experienced usually after work.

### 2.6. Sampling technique

#### 2.6.1. Inclusion criteria.

Male and female bakery workers aged between 18 and 65 years with 1 or more years of working experience.

#### 2.6.2. Exclusion criteria.

Male and female bakery workers aged below 18 years and above 65 years without 1 to 2 years of working experience.

#### 2.6.3. Specimen collection.

An aseptic venipuncture Blood collection method was employed by the researchers to collect 5 mL of blood samples with the use of needles and syringes from the subjects at the end of their shifts. The blood samples were collected into EDTA bottles to prevent blood coagulation by chelating the calcium present in the blood (Chelate), which was transported into the Medical Laboratory in a cold chain, to preserve sample integrity. The blood samples were then subjected to Haematological parameter testing using Sysmex auto-analyzer and manual method for ESR estimation.

### 2.7. Laboratory analysis

#### 2.7.1. Full blood count.

An estimated full blood count was carried out using haematological analyzer (Sysmex-2000) in the Haematology unit, at Kwara State University, Malete.

### 2.8. ESR (Westergren method)

#### 2.8.1. Procedure of ESR test.

The EDTA blood samples were well mixed by manually inverting the tube 10 times before transferring the sample. The Westergren method was done by transferring 2 mL of potassium-EDTA blood into a vial containing a defined amount of 3.8% sodium citrate (0.2 mL) solution. The blood was mixed thoroughly by manually inverting 10 times. The 150-mm graduated Westergreen tubes were filled with the blood sample levels to zero. The filled Westergreen tubes were stood in a Westergreen rack for 60 minutes, the level of fall red blood cells was measured exactly at 60 minutes ± 1 minute using a timer, and results were reported in mm/h.

## 3. Results

Table 1 shows the demographic of the study participants and control. A total of 60 bakery workers and 60 control samples were employed. Most of the study participants were between the ages of 18 and 45 years 53 (88.3%) and only 7 (11.6%) were above 45 years old. The majority of the study respondents were male 57 (95.5%) and only 3 (5%) were female. Most of the participants were single 41 (68.3%) and only 18 (31.6%) were married. The study was dominated by tertiary-level educated individuals 41 (68.3%) while secondary school-level educated only 19 (31.6%). The majority of the respondents were living in urban areas 48 (80.0%) while only 12 (20.0%) were living in rural areas.

**Table 1:** Sociodemographic characteristics of the study participants.

	Test (n = 60)	%	Control (n = 20)	%
Age				
18–45	53	88.3	14	70
>45	7	11.6	6	30
Gender				
Male	57	95	17	85
Female	3	5	3	5
Marital status				
Single	41	68.3	20	100
Married	19	31.6	0	0
Educational level				
Secondary	19	31.6	13	65
Tertiary	41	68.3	7	35
Residence				
Rural	48	80	18	90
Urban	12	20	2	10
Years of work experience				
1–10 years	52	86.6		
>10years	8	13.3		

In the control population, the highest number of participants (42) belonged to the age group 18 to 45 years (70%) while those aged above 45 years (18) had the lowest number of participants (30%). The majority of the control subjects (51) were male (85%) and only 9 were female (15%).

Table 2 shows health-related symptoms experienced by the participants.

**Table 2:** Medical history of the participants.

	Frequency	%
Dry skin		
Yes	28	46.6
No	32	53.3
Chill		
Yes	18	30
No	42	70
High body temperature		
Yes	12	20
No	48	80
Nausea		
Yes	09	15
No	51	85
Fatigue		
Yes	12	20
No	48	80
Muscle cramp		
Yes	5	91.6
No	55	8.3

Most of the study participants did not have dry skin 32 (53.3%) while 28 of the participants had complaints related to dry skin (46.6%). The majority (48 respondents) of the study participants complained of high body temperature 48 (80.0%) while only 12 (20%) of the participants complained of high body temperature. Respondents who had chills-related complaints were only 18 (30) while 48 (70%) had no chills-related complaints.

Table 3 shows the effect of heat on haematological parameters and ESRs of bakery workers.

**Table 3:** Effect of heat on haematological parameters and erythrocyte sedimentation rates of bakery workers (Mean ± SD).

Parameters	Test (n = 70)	*P* value	Control (n = 20)
ESR (mm/h)	2.13 ± 1.28	.984	10.05 ± 4.95
RBC(×10^12^/L)	6.708 ± 2.08	.975	5.46 ± 1.12
WBC(×10^9^/L)	7.425 ± 1.74	.981	6.95 ± 2.49
HB (g/dL)	15.535 ± 2.24	.904	15.31 ± 1.82
PCV (%)	47.597 ± 5.29	.888	44.02 ± 6.42
MCV (fl)	80.940 ± 11.64	1.00	82.59 ± 6.18
MCH (ng)	39.762 ± 7.92	.983	30.12 ± 4.66
MCHC (g/dL)	42.683 ± 8.64	.917	36.98 ± 2.38
PLT(×10^9^/L)	581.43 ± 94.39	.972	460.40 ± 154.90
LYM (%)	39.692 ± 5.26	.779	46.65 ± 14.85
GRAN%	55.452 ± 12.04	.999	49.61 ± 11.71

*P* value > .05 not significant.

*P* value < .05 Significant.

ESR = erythrocyte sedimentation rate, HB = haemoglobin, MCH = mean corpuscular haemoglobin, MCHC = mean corpuscular haemoglobin concentration, MCV = mean corpuscular volume, PCV = packed cell volume, PLT = platelet, RBC = red blood cell count, WBC = total white blood cell count.

The mean ± SD of the ESR and selected haematological parameters of bakery workers and control were computed, the ESR of bakery workers was found to be nonsignificantly lower (2.13 ± 1.28) compared to the control (10.05 ± 4.95). The majority of the selected haematological parameters among the bakery workers were found to be high compared to the control group; the mean ± SD for the RBC was nonsignificantly increased (6.708 ± 2.08) compared to the control group (5.46 ± 1.12); the mean ± SD for the total white blood cell (TWBC) was also found to be nonsignificantly higher (7.425 ± 1.74) compared to the control population (6.95 ± 2.49). The Hb was found to be moderately increased (15.53 ± 2.24) compared to the control group (15.31 ± 1.82); the mean ± SD for the PCV was also found to be slightly increased (47.597 ± 5.29) compared to the control subjects (44.02 ± 6.42). The Mean Cell Volume (MCV) was slightly lower (80.940 ± 11.64) compared to the control group (82.59 ± 6.18); conversely, the mean ± SD for the MCH was found to be nonsignificantly higher (39.762 ± 7.92) compared to the control population (30.12 ± 4.66). The MCHC was also found to be nonsignificantly raised (42.683 ± 8.64) when compared to the control group (36.98 ± 2.38); the mean ± SD for the PLT was also found to be nonsignificantly increased (581.43 ± 94.39) compared to the control population (460.40 ± 154.90). The Lymphocyte (LYM) was slightly lower (39.69 ± 5.26) compared to the control group (46.65 ± 14.85), the mean ± SD for the GRAN was found to be nonsignificantly raised (55.45 ± 12.04) compared to the control subjects (49.61 ± 11.71).

Table 4 shows the effect of the year of work experience on selected haematological parameters and ESR of bakery workers.

**Table 4:** Effect of year of work experience on selected haematological parameters and erythrocyte sedimentation rate of bakery workers.

Parameters	(1–10) years	(>10) years	*P* value
ESR (mm/h)	1.97 ± 1.36	2.52 ± 1.59	.08
RBC(×10^12^/L)	6.38 ± 2.65	4.83 ± 1.54	.02*
WBC(×10^9^/L)	6.94 ± 2.55	7.18 ± 2.32	1.0
HB (g/dL)	14.55 ± 4.49	14.42 ± 4.36	1.0
PCV (%)	44.76 ± 13.07	42.30 ± 8.93	.4
MCV (fl)	75.52 ± 23.3	81.23 ± 66.43	1.0
MCH (ng)	37.53 ± 12.71	33.47 ± 11.23	.5
MCHC (g/dL)	40.34 ± 13.7	34.95 ± 10.56	.2
PLT (×10^9^/L)	547.12 ± 173.78	514.75 ± 113.54	.4
LYM (%)	36.98 ± 11.16	40.52 ± 16.76	.5
GRAN (%)	52.79 ± 18.07	39.82 ± 14.34	.004*

ESR = erythrocyte sedimentation rate, HB = haemoglobin, MCH = mean corpuscular haemoglobin, MCHC = mean corpuscular haemoglobin concentration, MCV = mean corpuscular volume, PCV = packed cell volume, PLT = platelet, RBC = red blood cell count, WBC = total white blood cell count.

The mean ± SD of ESR and selected haematological parameters of bakery workers based on their year of work experience were computed. The ESR, TWBC, LYM, and MCV of bakery workers between 1 to 10 years of work experience were found to be nonsignificantly reduced (1.97 ± 1.36, 6.94 ± 2.55, 36.98 ± 11.16, and 75.52 ± 23.3) respectively, compared to that of bakery workers (2.52 ± 1.59, 7.18 ± 2.32, 40.52 ± 16.76, and 81.23 ± 666.43) with > 10 years of work experience. On the other hand, the RBC, PCV, MCHC, PLT and GRAN of bakery workers between 1 to 10years work experience were found to be high (6.38 ± 2.65, 44.76 ± 13.07, 40.34 ± 13.7, 547.12 ± 173.78, and 52.75 ± 18.07) respectively, compared to bakery workers > 10 years of work experience (4.83 ± 1.54, 42.30 ± 13.07, 514.75 ± 113.54, and 39.82 ± 14.34).

## 4. Discussion

The issue of occupational heat exposure is becoming increasingly pressing on a global scale due to the effects of climate change.^[1]^ Working in extreme heat (i.e., temperatures above 35°C), whether indoors or outdoors, puts workers’ health at risk and lowers labor productivity.^[1]^ Overexposure to heat is a highly dangerous occupational hazard, especially in indoor and outdoor environments without adequate temperature control.^[2]^ According to estimates, heat exposure costs South East Asian workers 20% of their working hours.^[3]^ Every cellular molecule’s kinetic energy increases when it is exposed to extreme heat continuously. The energy is subsequently dispersed throughout the cell. The possibility exists that heat-producing proteins may hydrolyze or accumulate inside cells and in many cellular organs, including the nucleus, mitochondria, and microbes.^[4]^

This study comprised a total number of 60 bakery workers and 20 control samples. Most of the study participants were between the ages of 18 and 45 years 53 (88.3%) and only 7 (11.6%) were above 45 years old. The majority of the study respondents were male 57 (95.5%) and only 3 (5%) were female. Most of the participants were single 41 (68.3%) and only 18 (31.6%) were married. The study was dominated by tertiary-level educated individuals 41 (68.3%) while secondary school-level educated only 19 (31.6%). The majority of the respondents were living in urban areas 48 (80.0%) while only 12 (20.0%) were living in rural areas.

In this study, Most of the study participants had dry skin 32 (53.3%) while 28 of the participants had complaints related to dry skin (46.6%). The majority of the study participants have complained of high body temperature 48 (80.0%) while only 12 (20%) of the participants have complained of high body temperature this agrees with the study of^[17]^ who found similar symptoms among heat stress workers. Respondents who had chills-related complaints were only 18 (30) while 48 (70%) had no chills-related complaints.

In this study the mean ± SD of ESR and selected hematology parameters of bakery workers and control were computed, and the ESR of bakery workers was found to be lower (2.13 ± 1.28) compared to (10.05 ± 4.95) of the control. In this study, the RBC was high (6.708 ± 2.08) compared to (5.46 ± 1.12) of the control group. The HB was found to be moderate (15.53 ± 2.24) compared to (15.31 ± 1.82) of the control group, the mean ± SD for the PCV was also found to be high (47.597 ± 5.29) compared to (44.02 ± 6.42) of the control subject, this study disagrees with the studies of^[17]^ and^[18]^ which reported lower values of RBC, HB, and PCV. In hot climates when humans are exposed to high ambient temperatures, haemo-concentrations are developed due to dehydration, asphyxia, or excitement, causing the release of erythrocytes in the spleen which can result in abnormally higher PCV levels, on the other hand, some authors^[19]^ reported that season did not affect the Hb concentration. The MCV, MCH, and MCHC were found to be high 80.940 ± 11.64, 39.762 ± 7.92, and 42.683 ± 8.64 respectively, compared to 82.59 ± 6.18, 30.12 ± 4.66, and 36.98 ± 2.38 of the control group respectively, this study aligned with the study of^[17]^ who also found high MCV, MCH, and MCHC among heat-stressed bakery workers. This study observed the mean ± SD for the Platelet to be high (581.43 ± 9439) compared to (460.40 ± 154.90) of the control population, this study disagrees with the study of^[18]^ who found low platelet value among bakery workers exposed to heat stress. The observation is compatible with a prior study showing that individual differences in platelet concentration persist (Hoareau et al, 2014). In this study the mean ± SD for the TWBC was found to also be high (7.425 ± 1.74) compared to (6.95 ± 2.49) of the control population, this contrasted with the study carried out on bakery workers by^[20]^ which reported that WBC value was lower in heat-stressed workers. The finding is also by the study of^[17]^ which also found high WBC in bakery workers. WBCs are crucial for the immune response, and higher counts could indicate an overactive immune system, suggesting the possibility of ongoing inflammation. The LYM was low (39.692 ± 5.26) compared to (46.65 ± 14.85) of the control group, this study is in accordance with the study of^[17]^ who also found low LYM value among heat stress bakery workers. In this study the mean ± SD for the granulocyte was found to be high (55.452 ± 12.04) compared to (49.61 ± 11.71) of the control subject, this study disagrees with the result obtained by^[21]^ who found the high value of granulocyte among heat stress bakery workers, but agrees with the study of^[17]^ who also found high granulocyte value in the among people in the hottest area. A higher granulocyte value has previously been associated with increased systemic inflammation and stress.^[18,20,22]^

This study found TWBC, LYM, and MCV of bakery workers between 1-10years of work experience were found to be low 6.94 ± 2.55, 36.98 ± 11.16, and 75.52 ± 23.3 respectively compared to 2.52 ± 1.59, 7.18 ± 2.32, 40.52 ± 16.76, and 81.23 ± 666.43 of bakery workers > 10 years of work experience respectively, this coincides with the study of^[20]^ who also found lower values of WBC, LYM, GRAN, and PCV among workers below 10 years compare to workers above 10 years of heat exposure and suggested that the greater the exposure period, the greater the effect on physiological variables of workers. On the other hand, the RBC, PCV, MCHC, PLT, and GRAN of bakery workers between 1-10years work experience were found to be high 6.38 ± 2.65, 44.76 ± 13.07, 40.34 ± 13.7, 547.12 ± 173.78, and 52.75 ± 18.07 respectively compared to 4.83 ± 1.54, 42.30 ± 13.07, 514.75 ± 113.54, and 39.82 ± 14.34 of bakery workers > 10 years of work experience.

## 5. Conclusion

The research focuses on both outdoor and indoor settings, offering insight into the risks encountered by workers, particularly in areas lacking efficient climate management. Occupational heat is a growing global problem due to its effects on health and productivity. The findings show that, in comparison to the control group, ESRs are lower in bakery workers. Although there are nonsignificant increases in certain haematological measures among bakery workers, the study raises the possibility of correlations between heat exposure and changes in blood parameters. The study also examines the impact of job experience on these variables, identifying subtle variations according to the length of heat exposure.

The study emphasizes how critical it is to comprehend how heat exposure affects hematology, especially in the case of bakery workers. The results establish the framework for further research into mitigating strategies and treatments to protect the health and well-being of workers in heat-prone situations and add to the expanding body of knowledge on occupational heat risks.

## 6. Recommendations

It is advised that preventative steps be taken to address the possible health effects of occupational heat exposure among bakery workers in light of the research findings.

To reduce exposure to heat, workplace measures should be put into place. This can entail better climate control systems, sufficient ventilation, planned pauses in chilly settings, and the provision of safety equipment, including cooling vests.

Furthermore, educational initiatives should be created to raise bakery employees’ knowledge of the warning signs and symptoms of heat-related illnesses. Training ought to stress the value of drinking enough water, taking rests in cool spots, and identifying the early warning symptoms of heat exhaustion.

Bakery employees should have a regular health checkup program that includes tracking haematological markers. This enables prompt intervention and aids in the early detection of any deviations from normal values.

Employees and management should also get regular training on the value of preserving a safe and healthy work environment. Guidelines for controlling workload during hot hours and fostering a culture of safety are two examples of this.

In addition, regulations about heat exposure at work must be established and implemented. This could entail establishing temperature thresholds, particularly indoors, and delineating particular procedures for intense heat waves.

To further explore the long-term health impacts of heat exposure on bakery workers, more research in this field is needed. Providing thorough standards for occupational heat management, may entail long-term research and working with medical experts.

## 7. Limitations

The cross-sectional design of this investigation presented limitations. The medical diagnosis of the symptoms linked to heat stress that was reported was not a factor in the study. The self-reported data used in this study was acquired straight from the bakery employees. Due to recall bias, several heat-related health problems may have been underreported based on self-reported heat stress-related symptoms. In this study, potential confounders like alcohol and smoking were not taken into consideration. Finally, because there were so few female participants in the study, it was not possible to examine the relationship between gender, work experience, and health symptoms.

## Author contributions

**Conceptualization:** Arinze Favour Anyiam.

**Data curation:** Arinze Favour Anyiam.

**Formal analysis:** Arinze Favour Anyiam.

**Investigation:** Arinze Favour Anyiam, Emmanuel Ifeanyi Obeagu.

**Methodology:** Arinze Favour Anyiam, Onyinye Cecilia Arinze-Anyiam, Ajiboye Mariam Oyindamola, Emmanuel Ifeanyi Obeagu.

**Resources:** Onyinye Cecilia Arinze-Anyiam, Ajiboye Mariam Oyindamola.

**Supervision:** Emmanuel Ifeanyi Obeagu.

**Validation:** Emmanuel Ifeanyi Obeagu.

**Writing – original draft:** Arinze Favour Anyiam, Onyinye Cecilia Arinze-Anyiam, Ajiboye Mariam Oyindamola, Emmanuel Ifeanyi Obeagu.

**Writing – review & editing:** Arinze Favour Anyiam, Onyinye Cecilia Arinze-Anyiam, Ajiboye Mariam Oyindamola, Emmanuel Ifeanyi Obeagu.
